# Supplementary material for: Trends in the estimated proportion of outpatients with menstrual disorders and the number of prescribed low-dose estrogen/progestin drugs in Japan: A descriptive study
Source: PLoS One. 2025 Jul 18;20(7):e0327774. doi: 10.1371/journal.pone.0327774 (PMC12273988; doi:10.1371/journal.pone.0327774)
Supplement: S1 File — This file contains a list of direct hyperlinks to the publicly available original datasets used for this study, including the Patient Survey, the Comprehensive Survey of Living Conditions, and the National Database (NDB) Open Data. (DOCX) [file pone.0327774.s001.docx]

Direct links to the original dataset

The patient Survey

Number of estimated patients

2023

https://www.e-stat.go.jp/stat-search/file-download?statInfId=000040234267&fileKind=1

2020

https://www.e-stat.go.jp/stat-search/file-download?statInfId=000032212014&fileKind=1

2017

https://www.e-stat.go.jp/stat-search/file-download?statInfId=000031790732&fileKind=1

2014

https://www.e-stat.go.jp/stat-search/file-download?statInfId=000031349524&fileKind=1

2011

https://www.e-stat.go.jp/stat-search/file-download?statInfId=000017030953&fileKind=1

2008

https://www.e-stat.go.jp/stat-search/file-download?statInfId=000006005682&fileKind=1

2005

https://www.e-stat.go.jp/stat-search/file-download?statInfId=000002410854&fileKind=1

2002

https://www.e-stat.go.jp/stat-search/file-download?statInfId=000002481121&fileKind=1

1999

https://www.e-stat.go.jp/stat-search/file-download?statInfId=000002488007&fileKind=1

Estimated population

2023

https://www.e-stat.go.jp/stat-search/file-download?statInfId=000040234541&fileKind=0

2020

https://www.e-stat.go.jp/stat-search/file-download?statInfId=000032212298&fileKind=0

2017

https://www.e-stat.go.jp/stat-search/file-download?statInfId=000031791009&fileKind=0

2014

https://www.e-stat.go.jp/stat-search/file-download?statInfId=000031349408&fileKind=0

2011

https://www.e-stat.go.jp/stat-search/file-download?statInfId=000020398227&fileKind=0

2008

https://www.e-stat.go.jp/stat-search/file-download?statInfId=000031803276&fileKind=0

2005

https://www.e-stat.go.jp/stat-search/file-download?statInfId=000031803296&fileKind=0

2002

https://www.e-stat.go.jp/stat-search/file-download?statInfId=000031803278&fileKind=0

1999

https://www.e-stat.go.jp/stat-search/file-download?statInfId=000031803298&fileKind=0

The Comprehensive Survey of Living Conditions

Number of people who reported symptoms

2022

https://www.e-stat.go.jp/stat-search/file-download?statInfId=000040071859&fileKind=1

2019

https://www.e-stat.go.jp/stat-search/file-download?statInfId=000031964400&fileKind=1

2016

https://www.e-stat.go.jp/stat-search/file-download?statInfId=000031595491&fileKind=1

2013

https://www.e-stat.go.jp/stat-search/file-download?statInfId=000025708590&fileKind=1

2010

https://www.e-stat.go.jp/stat-search/file-download?statInfId=000012658484&fileKind=1

2007

https://www.e-stat.go.jp/stat-search/file-download?statInfId=000002576947&fileKind=1

2004

https://www.e-stat.go.jp/stat-search/file-download?statInfId=000002344968&fileKind=1

2001

https://www.e-stat.go.jp/stat-search/file-download?statInfId=000002350230&fileKind=1

1998

https://www.e-stat.go.jp/stat-search/file-download?statInfId=000002352829&fileKind=1

Number of study population

2022

https://www.e-stat.go.jp/stat-search/file-download?statInfId=000040071598&fileKind=1

2019

https://www.e-stat.go.jp/stat-search/file-download?statInfId=000031964320&fileKind=1

2016

https://www.e-stat.go.jp/stat-search/file-download?statInfId=000031595435&fileKind=1

2013

https://www.e-stat.go.jp/stat-search/file-download?statInfId=000025708497&fileKind=1

2010

https://www.e-stat.go.jp/stat-search/file-download?statInfId=000012658508&fileKind=1

2007

https://www.e-stat.go.jp/stat-search/file-download?statInfId=000002577070&fileKind=1

2004

https://www.e-stat.go.jp/stat-search/file-download?statInfId=000002344932&fileKind=1

2001

https://www.e-stat.go.jp/stat-search/file-download?statInfId=000002352799&fileKind=1

1998

https://www.e-stat.go.jp/stat-search/file-download?statInfId=000002352799&fileKind=1

2015 Model population (for age standardization)

https://www.e-stat.go.jp/stat-search/file-download?statInfId=000040206105&fileKind=2

NDB Open data

2023

Oral, outpatient, pharmacy

https://www.mhlw.go.jp/content/12400000/001495390.xlsx

Oral, outpatient, hospital

https://www.mhlw.go.jp/content/12400000/001495396.xlsx

Oral, impatient

https://www.mhlw.go.jp/content/12400000/001495419.xlsx

2022

Oral, outpatient, pharmacy

https://www.mhlw.go.jp/content/12400000/001258655.xlsx

Oral, outpatient, hospital

https://www.mhlw.go.jp/content/12400000/001258660.xlsx

Oral, impatient

https://www.mhlw.go.jp/content/12400000/001258662.xlsx

2021

Oral, outpatient, pharmacy

https://www.mhlw.go.jp/content/12400000/001122626.xlsx

Oral, outpatient, hospital

https://www.mhlw.go.jp/content/12400000/001122628.xlsx

Oral, impatient

https://www.mhlw.go.jp/content/12400000/001122630.xlsx

2020

Oral, outpatient, pharmacy

https://www.mhlw.go.jp/content/12400000/001262339.xlsx

Oral, outpatient, hospital

https://www.mhlw.go.jp/content/12400000/001262341.xlsx

Oral, impatient

https://www.mhlw.go.jp/content/12400000/001262343.xlsx

2019

Oral, outpatient, pharmacy

https://www.mhlw.go.jp/content/12400000/000821759.xlsx

Oral, outpatient, hospital

https://www.mhlw.go.jp/content/12400000/000821764.xlsx

Oral, impatient

https://www.mhlw.go.jp/content/12400000/000821766.xlsx

2018

Oral, outpatient, pharmacy

https://www.mhlw.go.jp/content/12400000/000711129.xlsx

Oral, outpatient, hospital

https://www.mhlw.go.jp/content/12400000/000560110.xlsx

Oral, impatient

https://www.mhlw.go.jp/content/12400000/000560112.xlsx

2017

Oral, outpatient, pharmacy

https://www.mhlw.go.jp/content/12400000/000730485.xlsx

Oral, outpatient, hospital

https://www.mhlw.go.jp/content/12400000/000730488.xlsx

Oral, impatient

https://www.mhlw.go.jp/content/12400000/000730490.xlsx

2016

Oral, outpatient, pharmacy

https://www.mhlw.go.jp/content/12400000/000347790.xlsx

Oral, outpatient, hospital

https://www.mhlw.go.jp/content/12400000/000347792.xlsx

Oral, impatient

https://www.mhlw.go.jp/content/12400000/000347794.xlsx

2015

Oral, outpatient, pharmacy

https://www.mhlw.go.jp/file/06-Seisakujouhou-12400000-Hokenkyoku/0000177287.xlsx

Oral, outpatient, hospital

https://www.mhlw.go.jp/file/06-Seisakujouhou-12400000-Hokenkyoku/0000177289.xlsx

Oral, impatient

https://www.mhlw.go.jp/file/06-Seisakujouhou-12400000-Hokenkyoku/0000177291.xlsx

2014

Oral, outpatient, pharmacy

https://www.mhlw.go.jp/file/06-Seisakujouhou-12400000-Hokenkyoku/0000139841.xlsx

Oral, outpatient, hospital

https://www.mhlw.go.jp/file/06-Seisakujouhou-12400000-Hokenkyoku/0000139843.xlsx

Oral, impatient

https://www.mhlw.go.jp/file/06-Seisakujouhou-12400000-Hokenkyoku/0000139845.xlsx
