# Supplementary material for: Trends in the estimated proportion of outpatients with menstrual disorders and the number of prescribed low-dose estrogen/progestin drugs in Japan: A descriptive study
Source: PLoS One. 2025 Jul 18;20(7):e0327774. doi: 10.1371/journal.pone.0327774 (PMC12273988; doi:10.1371/journal.pone.0327774)
Supplement: Attachment — Submitted filename: Response to Reviewers v2.docx [file pone.0327774.s003.docx]

Dear Dr. Marcio Alexandre H. Rodrigues, Academic Editor, PLOS ONE,

Thank you for your thoughtful review of our manuscript and for the opportunity to revise our work. We appreciate the time and expertise that you and the reviewer have dedicated to providing constructive feedback. We have carefully addressed all comments and suggestions, and we believe the manuscript has been substantially improved as a result.

Below, we provide a point-by-point response to all comments, detailing the changes made to the manuscript. Changes are highlighted in the revised manuscript with track changes.

UPDATE REGARDING NEW DATA:

While this manuscript was under review, the 2022 and 2023 NDB Open Data, and 2023 Patient Survey became publicly available. we have updated the relevant sections of the manuscript, figures, and tables to incorporate these latest data (now covering 1999–2023 for the Patient Survey and 2014–2023 for NDB Open Data). The main findings and conclusions remain unchanged, but the Results section and Figures 1 and 3 have been revised to reflect the expanded dataset. All such changes are highlighted in the revised manuscript.

RESPONSES TO JOURNAL REQUIREMENTS:

1. Manuscript style requirements:

> Please ensure that your manuscript meets PLOS ONE's style requirements, including those for file naming.

We have carefully revised the manuscript to ensure it meets PLOS ONE's style requirements. Specifically, we have:

- Formatted the main body according to the journal template

- Ensured proper formatting of titles, authors, and affiliations

- Verified all file naming conventions follow PLOS ONE guidelines

2. Funding Statement:

> Please provide an amended statement that declares *all* the funding or sources of support received for the research submitted to the journal.

We have revised our Funding Statement as follows:

"This research was supported by Japan Society for the Promotion of Science (JSPS: https://www.jsps.go.jp/english/) Grant-in-Aid for Scientific Research (C) (Grant Number 23K01927 to MN) and Ministry of Health, Labour and Welfare of Japan (MHLW: https://www.mhlw.go.jp/stf/english/index.html) Health Labor Science Research Grant (Grant Number 23FB1002 to KN). There was no additional external funding received for this study."

3. Role of Funders:

> Please state what role the funders took in the study.

We have added the following statement:

"The funders had no role in study design, data collection and analysis, decision to publish, or preparation of the manuscript."

We included the statement above (Funding Statement and Role of Funders) in the cover letter.

4. Data Availability Statement reads:

> Unfortunately, the repository you have noted in your Data Availability statement does not qualify as an acceptable data repository according to PLOS's standards.

We appreciate this guidance. We have now uploaded our compiled dataset to figshare with the following DOI: 10.6084/m9.figshare.29148170. The dataset includes the processed data from all three government sources used in our analysis: the Patient Survey, Comprehensive Survey of Living Conditions, and the NDB Open Data. We have also compiled a detailed list of direct links to the original datasets (Patient Survey, Comprehensive Survey of Living Conditions, and NDB Open Data) as requested by the editorial office. These URLs are now included in the Data Availability Statement and in the supplementary materials.

Our revised Data Availability Statement reads:

"All data underlying the findings described in our manuscript are available from figshare (DOI: 10.6084/m9.figshare.29148170). The original raw datasets analyzed during the current study are available from e-Stat (https://www.e-stat.go.jp/en) for the Patient Survey and the Comprehensive Survey of Living Conditions, and from the Ministry of Health, Labour and Welfare (https://www.mhlw.go.jp/stf/seisakunitsuite/bunya/0000177182.html) for the NDB Open Data.

The original datasets analyzed in this study are publicly available from the following sources:

[list of URLs]"

5. Ethics Statement placement:

> Your ethics statement should only appear in the Methods section of your manuscript.

We have removed the ethics statement from other sections and ensured it appears only in the Methods section of the manuscript.

RESPONSES TO ACADEMIC EDITOR’S COMMENTS:

Comment #1:

> One of the most prominent concerns arises from the divergence between trends in self-reported symptoms and outpatient visits... This incongruity raises important questions.

Thank you for highlighting the important issue of the divergence between trends in self-reported menstrual symptoms and outpatient visits for menstrual disorders. We agree that this paradox is central to interpreting our findings. In the revised manuscript, we have expanded the Discussion section to address this point in greater depth.

Specifically, we now discuss that the observed increase in outpatient follow-up visits for menstrual disorders is unlikely to reflect a true rise in disease burden, as the prevalence of self-reported symptoms has remained stable or declined. Instead, we suggest that several social and systemic factors may have contributed to increased healthcare utilization. These include greater public awareness of menstrual health, reduced stigma surrounding gynecological consultation, improved access to medical care, and the expansion of insurance coverage for LEPs. Such factors may have encouraged more women to seek medical attention for symptoms that were previously self-managed or considered a normal aspect of menstruation. We have also cited recent literature on the impact of awareness, stigma, and health system changes on care-seeking behavior (e.g., Tanaka et al., 2013; Ohde et al., 2008).

The following paragraph has been added to the Discussion section:

“The divergence between the increasing rate of medical follow-ups and the stagnation or slight decline in self-reported symptoms may reflect changes in health-seeking behavior, increased public awareness, reduced stigma, and improved access to care. [15–18, 25, 26]”

Comment #2:

> The results indicate age-specific peaks in outpatient visits and symptom reporting, with notable shifts over time... However, the study does not offer a thorough explanation or control for external variables...

We acknowledge this limitation and have conducted additional age-standardized analysis to address this concern. We have added a new paragraph in the Methods, Results and the Discussion section:

Methods section:

“To account for changes in the age distribution of the female population over time, age-standardized rates were calculated for both the estimated proportion of outpatients with menstrual disorders and endometriosis (from the Patient Survey) and the proportion of persons reporting irregular menstruation or menstrual pain (from the Comprehensive Survey of Living Conditions). Age-specific rates were calculated for each 5-year age group, and the 2015 model female population in Japan was used as the standard population. The age-standardized rate for each survey year was obtained by weighting the age-specific rates by the corresponding age group proportions in the standard population and summing these weighted rates [24].”

Results section:

"After adjusting for changes in the age distribution of the female population, the age-standardized trends in the estimated proportion of outpatients with menstrual disorders and endometriosis were similar to the crude trends (see additional lines in Figure 1A–C). The increase in follow-up visits for menstrual disorders and endometriosis since 2014 remained evident after age standardization."

Discussion section:

“Importantly, age-standardized analyses showed that these trends remained evident even after adjusting for changes in the age distribution of the female population (see Figure 1A–C). This suggests that the recent increase in follow-up visits for menstrual disorders and endometriosis cannot be explained solely by demographic shifts, but likely reflects other factors such as changes in health-seeking behavior, insurance coverage, or public awareness.”

Comment 3:

> The observed 3.8-fold increase in LEP prescriptions from 2014 to 2021, particularly among younger women, is attributable to enhanced accessibility following expanded insurance coverage. Nonetheless, the results focus primarily on prescription volume and drug pricing, without addressing treatment adherence, efficacy, or patient-reported outcomes.

Thank you for this insightful comment. While our study design using aggregate government data cannot directly assess patient-reported outcomes, we acknowledge this limitation and have expanded our discussion to address these important considerations:

“Furthermore, because our analyses were based on aggregated data from government statistics, we were unable to assess individual-level treatment adherence, long-term persistence, symptom improvement, or patient-reported outcomes such as quality of life. Therefore, while we observed a substantial increase in LEP prescriptions and a reduction in economic burden due to generic drugs, we cannot directly determine whether these changes translated into improved clinical outcomes or long-term adherence. Future studies using individual-level data, such as health insurance claims or electronic medical records, are needed to clarify the real-world effectiveness of LEP treatment for menstrual disorders and the impact of generic drug introduction on adherence.”

Comment #3.1:

> Moreover, while drug utilization data are detailed, the lack of correlation with outpatient visit or symptom trends weakens the integrated understanding of treatment effectiveness.

Thank you for your comment regarding the relationship between LEP prescription volume and outpatient visit rates. We agree that these two indicators are derived from different data sources and represent different denominators and populations. Therefore, we believe that formal correlation analysis between these variables would not be statistically meaningful or scientifically appropriate. Instead, we have described the temporal trends of each indicator separately and highlighted their coinciding increases in the Results and Discussion sections. We hope this approach clarifies our intention to provide a descriptive overview rather than to imply a direct statistical association.

Comment #4:

> The discussion and conclusion sections present a well-structured narrative linking the increased use of LEPs to policy changes and access to affordable treatment. However, a deeper academic analysis raises important questions that were not sufficiently explored.

Thank you for this thoughtful observation. We have substantially expanded our Discussion section to include a more critical interrogation of potential contributing factors beyond insurance coverage:

“In addition to the expansion of insurance coverage for LEPs, several other factors may have contributed to the observed increase in follow-up visits for menstrual disorders. Recent studies have highlighted the importance of social and cultural factors, such as increased public awareness, reduced stigma surrounding menstruation, and evolving attitudes toward women's health, in influencing healthcare-seeking behavior among women [25, 33, 34]. In Japan, healthcare avoidance for menstrual symptoms has been associated with attitudes of endurance, limited health literacy, and lack of awareness about available treatment options [25]. Improvements in diagnostic capabilities and greater emphasis on menstrual health in public discourse may also have encouraged more women to seek medical care, even for symptoms previously considered normal. These findings are consistent with international literature showing that social context, education, and economic factors can significantly affect access to and utilization of menstrual health services [33, 34]. Therefore, while the increased use of LEPs and insurance coverage are important, these broader social and systemic changes should also be considered when interpreting recent trends.”

Comment #4.1:

> Moreover, the authors note a discrepancy between increasing medical follow-ups and the stagnation or slight decline in self-reported symptoms; however, this paradox is only superficially addressed.

Thank you for your comment regarding the paradox between increasing medical follow-ups and the stagnation or decline in self-reported symptoms. As noted in our response to Comment #1, we have expanded the Discussion to address this inconsistency in detail.

We now discuss that this divergence may reflect a combination of increased recognition of menstrual disorders as legitimate medical conditions, reduced stigma, and improved access to care, rather than a true increase in symptom prevalence. We also note that socioeconomic and educational factors may influence who seeks treatment, and that further qualitative research is needed to clarify these mechanisms. Relevant literature has been cited to support these interpretations (e.g., Tanaka et al., 2013; Ohde et al., 2008; Getahun SB et al., 2023).

The following paragraph has been added to the Discussion section:

“The divergence between the increasing rate of medical follow-ups and the stagnation or slight decline in self-reported symptoms may reflect changes in health-seeking behavior, increased public awareness, reduced stigma, and improved access to care. [15–18, 25, 26]”

Comment #5:

> Minor revisions: How can future studies address the inability to distinguish between primary and secondary dysmenorrhea in aggregated data?

Thank you for your insightful comment regarding the inability to distinguish between primary and secondary dysmenorrhea in aggregated data. We agree that this is an important limitation. In the Japanese Patient Survey, cases of dysmenorrhea due to endometriosis or adenomyosis that are diagnosed during follow-up are reclassified under "endometriosis" or other relevant categories, and thus are excluded from the "menstrual disorders" category in subsequent surveys. Therefore, among follow-up outpatients, a certain proportion of secondary dysmenorrhea may be excluded. However, it is possible that undiagnosed cases of secondary dysmenorrhea remain within the "menstrual disorders" category, especially at earlier stages of care. As a result, complete differentiation between primary and secondary dysmenorrhea is not feasible using aggregated survey data. Future studies using individual-level data, such as health insurance claims or electronic medical records, would enable more precise classification.

The revised Discussion includes the following paragraph:

“Finally, because the aggregated data used in this study do not allow for the distinction between primary and secondary dysmenorrhea, it is possible that some cases of secondary dysmenorrhea, such as undiagnosed endometriosis or adenomyosis, may remain included in the "menstrual disorders" category, while diagnosed cases are reclassified under "endometriosis" or other relevant categories. Therefore, a certain proportion of secondary dysmenorrhea may be excluded from the follow-up group, but complete differentiation is not possible. Future research using individual-level data is needed to address this issue.”

Comment #5-1:

> What are the underlying reasons why many women still opt for over-the-counter (OTC) medications instead of seeking medical treatment for menstrual disorders?

Thank you for your insightful question regarding the underlying reasons why many women still opt for over-the-counter (OTC) medications instead of seeking medical treatment for menstrual disorders.

Multiple factors contribute to this phenomenon, as supported by the literature and our own findings. First, OTC medications such as NSAIDs are easily accessible, affordable, and familiar, making them a convenient first-line option for many women, especially those with mild or moderate symptoms. Second, social and cultural factors—including the perception that menstrual pain is a normal experience, stigma surrounding gynecological visits, and reluctance to discuss menstruation—can discourage women from seeking professional care. Third, practical barriers such as time constraints, clinic hours, and the perceived burden of medical visits may also play a role. Fourth, some women may lack awareness of the availability or effectiveness of prescription treatments such as LEPs, or may have concerns about the safety or side effects of hormonal therapy. Economic considerations, even in the context of insurance coverage, can also influence treatment choices, particularly for students and young women.

These factors are consistent with previous studies in Japan and other countries (e.g., Tanaka et al., 2013; Ohde et al., 2008; Health and Global Policy Institute, 2018). We have added a brief summary of these points to the Discussion section.

In addition to the factors previously mentioned, younger women in Japan often face particular barriers to seeking gynecological care. Many perceive gynecology clinics as places primarily for pregnant or older women, and feel embarrassed or anxious about visiting for menstrual symptoms alone. Cultural norms and lack of comprehensive sex education further reinforce the idea that menstruation and reproductive health are private or even taboo topics, making it especially difficult for adolescents and young adults to seek professional help. These barriers are well-documented in recent studies and surveys of Japanese women . We have added this point to the Discussion section.

“Despite the availability of effective prescription treatments, many women continue to rely on OTC medications for menstrual pain. This preference may be attributed to the convenience, accessibility, and affordability of OTC drugs, as well as practical or economic barriers to clinic visits. [15–18]”

Comment #5-2:

> How significant is the reduction in economic burden due to generic drugs, and how does this affect long-term adherence to treatment?

Thank you for your comments regarding the evaluation of treatment adherence, clinical outcomes, and the impact of generic drugs on long-term adherence. As both comments (#3 and this) point out, our study was based on aggregated data and therefore could not directly assess individual-level adherence, persistence, or patient-reported outcomes. We have revised the Limitation section to clarify this point and to note that future research using individual-level data will be necessary to address these important questions.

Comment #5-3:

> Are there cultural stigmas or societal norms in Japan that discourage women from visiting healthcare providers for menstrual-related symptoms?

Thank you for your important question regarding cultural stigmas and societal norms in Japan that may discourage women from visiting healthcare providers for menstrual-related symptoms.

There is substantial evidence that cultural and social factors play a significant role in healthcare-seeking behavior for menstrual disorders in Japan. Menstruation remains a sensitive and often stigmatized topic; “period shame” and the expectation to conceal menstruation are widespread. Surveys and qualitative studies have shown that many Japanese women, especially adolescents and young adults, feel embarrassed to discuss menstruation or to be seen purchasing menstrual products. This stigma is reinforced by euphemistic language, social norms that encourage women to endure pain as a normal part of being female, and limited comprehensive sex education.

Additionally, there is a perception that gynecology clinics are primarily for pregnancy or older women, making it particularly difficult for younger women to seek care for menstrual symptoms. Studies have shown that a significant proportion of women with moderate to severe symptoms do not seek medical care, often citing embarrassment, normalization of pain, or the belief that their symptoms are not serious enough to warrant a visit. The lack of open discussion about menstruation in families, schools, and society at large further perpetuates these barriers.

We have added a brief summary of these points to the Discussion section, supported by recent literature.

“Cultural stigma and societal norms in Japan also contribute to the low rate of healthcare-seeking for menstrual disorders. Menstruation is often considered a private or taboo topic, and many women—especially adolescents and young adults—feel embarrassed to discuss menstrual symptoms or to visit a gynecologist for these issues. The expectation to endure menstrual pain as a normal part of womanhood, combined with limited reproductive health education and the perception that gynecology clinics are primarily for pregnancy or older women, further discourages medical consultation. These cultural and societal barriers have been well documented and are thought to play a significant role in healthcare avoidance for menstrual symptoms in Japan [15–18, 25, 26].”

Comment #5-4:

> Could the insurance coverage for LEPs be extended to include other menstrual-related conditions, and what would be the potential impact?

Could the insurance coverage for LEPs be extended to include other menstrual-related conditions, and what would be the potential impact?

Response:

Thank you for this important question. In Japan, LEPs are currently covered by public health insurance only for dysmenorrhea and endometriosis, but not for other menstrual-related conditions such as abnormal uterine bleeding, premenstrual syndrome (PMS), or polycystic ovary syndrome (PCOS). Expanding insurance coverage to include these additional indications could potentially improve access to effective treatment for a broader population of women and reduce the burden of untreated menstrual symptoms. Internationally, LEPs and similar combined oral contraceptives are used for a range of menstrual disorders, and their efficacy and safety for conditions such as abnormal uterine bleeding and PMS are well documented.

If insurance coverage were extended, more women with various menstrual-related conditions could benefit from medical management, which may lead to improved quality of life, reduced absenteeism, and possibly lower long-term healthcare costs due to better symptom control and prevention of complications. However, it is also important to consider potential challenges, such as increased healthcare expenditure for the public insurance system and the need for clear clinical guidelines to ensure appropriate use.

We have added a brief discussion of this point to the revised manuscript.

“In Japan, LEPs are currently covered by public health insurance only for dysmenorrhea and endometriosis [35]. However, LEPs have demonstrated efficacy for other menstrual-related conditions, such as abnormal uterine bleeding, premenstrual syndrome, [36–38] and polycystic ovary syndrome. If insurance coverage were expanded to include these indications, it could improve access to effective treatment for a wider range of women and potentially reduce the burden of untreated menstrual symptoms. Such a policy change may lead to improvements in quality of life and productivity, but would also require careful consideration of healthcare costs and the development of appropriate clinical guidelines to ensure rational use.”

Comment #5-5:

> How can the data from this study be used to predict future trends in menstrual health and improve healthcare accessibility for younger women?

Thank you for this insightful question. The multi-source national data used in our study provide a valuable foundation for monitoring and forecasting trends in menstrual health in Japan. By analyzing long-term changes in outpatient visits, self-reported symptoms, and prescription patterns, we can identify shifts in healthcare-seeking behavior, treatment uptake, and unmet needs among women—especially younger age groups. For example, the sharp increase in LEP prescriptions and follow-up visits among women in their 20s suggests greater awareness and access, but the persistent reliance on OTC medications and the decline in self-reported symptoms highlight ongoing barriers and possible under-recognition of menstrual health issues.

These data can inform predictive modeling of future demand for menstrual health services, guide resource allocation, and support the development of targeted interventions. For younger women, the age-stratified trends and identification of barriers (such as cost, stigma, and lack of reproductive health education) can help policymakers and healthcare providers design tailored outreach, education, and support programs. Furthermore, integrating these national statistics with emerging data sources—such as digital health records or menstrual tracking apps—could enable more precise, real-time monitoring and forecasting of menstrual health trends (see, e.g., Li K et al., and Hennegan J et al.).

We have added a summary of these points to the Discussion section.

“The national survey data used in this study provide a foundation for monitoring and predicting future trends in menstrual health in Japan. By tracking changes in outpatient visits, self-reported symptoms, and prescription patterns over time, it is possible to identify emerging needs and gaps in care, particularly among younger women. The observed increase in LEP prescriptions and follow-up visits among women in their 20s suggests improved access, but the continued reliance on OTC medications and the stagnation in self-reported symptoms indicate that barriers such as cost, stigma, and lack of health literacy persist. These findings highlight the importance of ongoing surveillance and the need for targeted interventions—such as health education, outreach, and policy reforms—to further improve healthcare accessibility and menstrual health outcomes for younger women. Future studies could also leverage digital health data and predictive modeling to better forecast demand and inform resource allocation [39, 40].”

Reviewers' comments:

Reviewer #1: An interesting work on the part of the authors. However, in my opinion, the study has two major problems:

1. The design of the study includes two research questions:

First question: Why the proportion of outpatients with menstrual disorders obtained from the Patient Survey (1999-2020) diverges from the proportion of persons reporting irregular menstruation or menstrual pain, obtained from the Comprehensive Survey of Living Conditions (1998-2022)? The answer to this question could be developed in a separate article. This would lead to the literature on self-perceived health status and the psychocultural conditioning factors that determine it. Moreover, to explore those conditioning factors from a women’s health perspective could be a promising research line.

Second question: Is the prevalence of menstrual disorders (regardless of the database used) and the prescription of LEPs obtained from NDB Open Data statistically correlated? Are there are clinical and epidemiological reasons to assume so? in my opinion the article should better justify the reason why this association is assumed.

If the research question is the first one, this implies a completely different article. Conversely, if the research question is the second one, I don’t see the reason why to use two different datasets to infer the prevalence of menstrual disorders.

Thank you for your thoughtful comments regarding the research questions and study design. We agree that our study addresses two related but distinct descriptive aims:

(1) to compare trends in the estimated proportion of outpatients with menstrual disorders (from medical facility-based Patient Survey data) and the proportion of persons reporting irregular menstruation or menstrual pain (from population-based Comprehensive Survey of Living Conditions), and

(2) to describe the trends in LEP prescriptions (from NDB Open Data) and examine their temporal relationship with changes in outpatient visits for menstrual disorders.

The rationale for using these multiple datasets is to provide a comprehensive, multi-faceted view of menstrual health trends in Japan from both medical care utilization and self-reported symptom perspectives. The divergence observed between outpatient visits and self-reported symptoms is itself a novel and important descriptive finding, suggesting changes in health-seeking behavior, awareness, or access to care, which we discuss in the revised Discussion section.

Regarding the second point, we did not perform statistical correlation analyses between prevalence and prescriptions, as the data sources, definitions, and units are different and not directly comparable. Instead, we present these trends descriptively and discuss their possible relationships and implications. We have revised the Introduction and Discussion to clarify the descriptive nature and objectives of the study, and to avoid over-interpreting associations between the datasets. We also emphasize the limitations of using aggregate data and the inability to draw causal or statistical inferences.

We hope these clarifications address your concerns and have revised the manuscript accordingly (see Introduction and Discussion).

Introduction section:

“The objectives of this descriptive study are twofold: first, to compare trends in the estimated proportion of outpatients with menstrual disorders (using medical facility-based data) and the proportion of persons reporting irregular menstruation or menstrual pain (using population-based self-report data); and second, to describe changes in LEP prescription patterns and their temporal relationship with outpatient visits for menstrual disorders. By integrating multiple national datasets, we aim to provide a comprehensive overview of menstrual health trends in Japan from both clinical and population perspectives.”

Discussion section:

“This study is descriptive in nature and does not attempt to establish statistical correlations or causal relationships between the prevalence of menstrual disorders and LEP prescriptions. The use of multiple datasets allows us to highlight differences and potential gaps between self-reported symptoms and medical care utilization, but the data sources are not directly comparable, and the observed temporal associations should be interpreted with caution. Further research using individual-level or linked data would be needed to explore these relationships in more detail.”

2. The article claims that no studies have provided reliable statistical information on the extent to which women with menstrual symptoms visit their doctors and are prescribed LEPs. However, according to the authors the study is purely descriptive. I don’t see reliable statistics. In fact, the usage of publicly available aggregate data and the absence of a minimal statistical analysis reduces the level of evidence that supports the conclusions. I think the authors made a significant effort to investigate, aggregate and describe the different sources of data related to the menstrual disorders and that is the contribution of the article.

Thank you for your thoughtful comments and for recognizing the contribution of our descriptive approach in aggregating and presenting national data on menstrual disorders and LEP prescriptions. We fully agree that our study is descriptive in nature and does not provide inferential statistical analysis or high-level evidence in the sense of randomized controlled trials or analytic epidemiological studies. Our intention in using the term "reliable statistical information" was to emphasize that our findings are based on large-scale, government-collected, nationally representative datasets, which provide a comprehensive overview of trends in menstrual health and healthcare utilization in Japan over two decades.

However, we recognize that the level of evidence is inherently limited by the use of aggregated, publicly available data and the absence of individual-level or causal analyses. To address this, we have revised the Introduction, Abstract, and Discussion to clarify the descriptive nature and limitations of our study, and to avoid overstating the strength of our conclusions. We now explicitly state that our findings should be interpreted as descriptive trends, and that further analytic studies are needed to establish causal relationships or more detailed statistical associations.

We appreciate your recognition that the aggregation and synthesis of these diverse data sources represent a valuable contribution to the literature, and we have revised the manuscript to more accurately reflect the scope and limitations of our work.

Abstract:

“These findings provide a descriptive overview of national trends in menstrual health and healthcare utilization, but should be interpreted with caution given the limitations of aggregate data and the absence of individual-level or inferential statistical analysis.”

Introduction section:

“The objectives of this descriptive study are twofold: first, to compare trends in the estimated proportion of outpatients with menstrual disorders (using medical facility-based data) and the proportion of persons reporting irregular menstruation or menstrual pain (using population-based self-report data); and second, to describe changes in LEP prescription patterns and their temporal relationship with outpatient visits for menstrual disorders. By integrating multiple national datasets, we aim to provide a comprehensive overview of menstrual health trends in Japan from both clinical and population perspectives.”

Discussion section:

“Nevertheless, the use of multiple national datasets in this study was intended to provide a comprehensive, descriptive overview of menstrual health trends in Japan from both clinical (medical facility-based) and population (self-reported) perspectives. Because the data sources, definitions, and units differ, no statistical correlation or causal inference was attempted between the prevalence of menstrual disorders and LEP prescriptions. Rather, we sought to highlight temporal trends and potential gaps between self-reported symptoms and medical care utilization. This descriptive approach allows for the identification of emerging needs and changes in health-seeking behavior, but the observed associations should be interpreted with caution. Although these limitations could have been overcome by conducting a more detailed study using more complete data, such as health insurance claims data, this study was conducted as a pilot study, giving priority to simplicity and using only publicly available data to examine trends in the indicators of menstruation-related diseases.”
